# Parenting interventions for families with refugee backgrounds: a randomized factorial, mixed-methods design study protocol

**DOI:** 10.1186/s13063-021-05766-9

**Published:** 2021-11-11

**Authors:** Joshua Patras, Merete Saus, Marcela Douglas, Ragnhild Bjørknes, Siri Gammelsæter, Lene-Mari Potulski Rasmussen, Therese Halvorsen, Ida Mari Haug, Ragnhild Risholm, Tuva Øktedalen, Reidar Jakobsen, Simon Peter Neumer

**Affiliations:** 1 UiT The Arctic University of Norway, Tromsø, Norway; 2 The Center for Child and Adolescent Mental Health – Eastern and Southern Norway, Oslo, Norway; 3 NORCE, Bergen, Norway; 4 University of Bergen, Bergen, Norway; 5 Norwegian University of Science and Technology, Trondheim, Norway; 6 Bufetat – Region South, Drammen, Norway

**Keywords:** Parenting intervention, Refugee families, Randomized factorial design, Measurement feedback, Mixed-methods, Ethnic minorities, Cultural adaptation, Effectiveness, Evidence-based practice, Parent practices

## Abstract

**Background:**

Raising children in new social and cultural contexts can be challenging for parents. In order to help parents address these challenges, the Norwegian government has instituted a policy of mandatory parent training for families who settle in Norway as refugees. The Incredible Years (IY) and The International Child Development Programme (ICDP) have been widely adopted throughout Norway. They have similar aims: to improve parenting through positive parenting practices and development of attachment behaviors. We will evaluate the use of these programs and a measurement feedback system (MFS) to give regular feedback to interventionists about parents’ progress during the course of the parenting intervention.

**Methods:**

The study is a mixed method, randomized factorial design aimed at evaluating the effect of parenting interventions and the use of feedback to address parental stress, child behavior, resilience, and parents’ mental health. Factor 1 is based on random assignment to one of the parenting interventions IY or ICDP. The parenting interventions are delivered over 15 weeks (IY) or 12 weeks (ICDP) in group-based settings. Factor 2 is based on random assignment of the parenting groups to the (a) with MFS or (b) without MFS condition. The MFS is answered weekly via a phone app, MittEcho, and results are sent to group leaders in the MFS condition. Additionally, the study explores the experiences of families settling in a new cultural context and participating with parenting programs via qualitative interviews. Participants will be recruited from a population of parents with children between the age of 6 and 12 years who settled in Norway as refugees within the previous 9 years. The target sample size is *N* = 360; *n* (IY) = 180, *n* (ICDP) = 180 families. This study is a collaboration between first-line, municipal services; their national governing agencies; family representatives; and a national network of research organizations.

**Discussion:**

This study endeavors to provide information about what helps families with refugee background integrate successfully into new cultural contexts with different laws, norms, and expectations. Whether or not these interventions can help to normalize this experience, reduce stress, and provide parents with new tools to improve their parenting and the lives of their children are important questions which we address. These findings can lead to the further establishment of evidence-based practices in Norway.

**Trial registration:**

ISRCTN35008070. Registered on February 24, 2020

## Administrative information

Note: the numbers in curly brackets in this protocol refer to SPIRIT checklist item numbers. The order of the items has been modified to group similar items (see http://www.equator-network.org/reporting-guidelines/spirit-2013-statement-defining-standard-protocol-items-for-clinical-trials/).

**Table:** 

Title {1}	Parenting interventions for families with refugee backgrounds: A randomized factorial, mixed-methods design study protocol
Trial registration {2a and 2b}.	ISRCTN35008070, ISRCTN All items from the WHO Trial Registry Data Set can be found herein.
Protocol version {3}	Protocol version 2: February, 3, 2020
Funding {4}	Kavli Trust (external funder) UiT The Arctic University of Norway (main project funder) Norwegian University of Science and Technology Regional Center for Child and Adolescent Mental Health – East and South NORCE Bufetat – Region South
Author details {5a}	^1^ UiT The Arctic University of Norway; Tromsø, Norway ^2^ The Center for Child and Adolescent Mental Health – Eastern and Southern Norway; Oslo, Norway ^3^ NORCE; Bergen, Norway ^4^ Norwegian University of Science and Technology; Trondheim, Norway ^5^ Bufetat – Region South; Drammen, Norway ^6^ University of Bergen, Norway ^7^ Corresponding author
Name and contact information for the trial sponsor {5b}	Monica Martinussen, monica.martinussen@uit.no
Role of sponsor {5c}	The sponsors and funders do not have an influence in study design, collection, management, analysis, nor interpretation of the results beyond initial approval of the protocol. Day-to-day running of the project, data collection, analysis, and reporting results are the responsibility of the protocol authors.

## Introduction

### Background and rationale {6a}

Raising children in new countries is challenging for most parents [1, 2]. Parents with refugee background have additional pressures to the usual stress that parents face [3]. These challenges could be related to war exposure [4], acculturation [5], poor mental health, and reduced family functioning [6]. These factors affect parenting differently [4], but they are seen as risk factors for child maladjustment [6]. Positive parenting coping strategies are demonstrated as protective factors to these child maladjustment risks [7, 8]. Parenting interventions such as the Incredible Years (IY) and the International Child Development Program (ICDP) may be uniquely positioned to address this challenge. The Incredible Years has been successfully adopted in many cultures and contexts [9]. Furthermore, IY has shown positive results in randomized trials with selected populations [10], though never with refugee populations. ICDP is a universal parent training program used in over 30 countries worldwide since the 1990s. The International Child Development Program (ICDP) has evidence of effect from other countries [11–15], but not from this particular population in Norway [16]. Both of these programs are widely implemented in social welfare and refugee services throughout Norway. Both of these interventions involve a group-based environment to learn proactive parenting skills to improve the family climate and give parents positive skills to help guide their children. The present study will provide knowledge regarding the effectiveness of these parenting interventions for families with refugee background who are integrating into Norwegian culture.

There has also been a growing need for improving treatment quality using measurement feedback systems (MFS) during treatment in mental health services [17]. It is believed that MFS can improve treatment quality by offering regular feedback to interventionists concerning the well-being of their clients. There are no MFS known to the project team that expressly addresses the needs of professionals working with group-based parenting interventions. To address this need, the project team developed an app that participants can access from their phone to answer a series of questions about their experience with the parenting interventions. The app, MittEcho, was developed to address this population and these interventions.

This study will have a mixed method, randomized factorial design that will include surveys, video observations, and interviews. Mixed methods refers to the use of qualitative and quantitative data collected together to address the primary research questions. The qualitative interviews will be conducted to gather information about the experiences of parents, children, and interventionists with relation to the overall questions of the utility and effectiveness of the interventions. The quantitative surveys will allow for the statistical testing of the primary and secondary outcomes. The multi-factorial design is a means to evaluate the parenting interventions (factor 1) and assess whether the addition of the MFS (factor 2) improves overall outcomes.

This study employs a multi-informant approach with data obtained from parents, children, teachers, and program group leaders. User perspectives and satisfaction will be explored throughout the project-period and used to adjust and adapt study procedures. Although IY and ICDP are implemented throughout Norway, the effectiveness of the programs for refugee families has not been established. This study, therefore, builds upon existing infrastructure to support these interventions and provides new, useful information for policymakers regarding the effectiveness of these programs. This can lead to the establishment of evidence-based practices in Norway.

### Objectives {7}

The primary aims of the present study are to evaluate the effectiveness of the IY and ICDP parenting interventions for improving primary outcome measures of parenting skills, lowering parental stress, and reducing problem behavior in children. The parenting programs are expected to have similar outcomes. Furthermore, the present study is an evaluation of the effectiveness of systematic, measurement feedback to improve treatment outcomes for refugee parents; therefore, we expect better outcomes for families whose group leaders are receiving systematic feedback about their group’s progress. This is a mixed method inquiry to evaluate whether IY and ICDP are appropriate interventions to address the challenges refugee parents experience while raising children in a new cultural context. We will conduct surveys and interviews of parents attending IY and ICDP groups and interviews with some of their children. The IY and ICDP group leaders will also be invited to fill out surveys and interviews. This multi-informant, multi-method approach provides different perspectives on the effectiveness of the interventions and the experiences of parents and practitioners. The interviews will contribute to better, overall knowledge of the usefulness of IY and ICDP for refugee parents with focus on who benefits the most from the interventions. Additionally, we will evaluate videos of IY sessions for analysis of the group dynamics, how families interact, and how they experience a group-based parenting intervention. IY collects video recordings as part of standard procedure, these recordings are not available for ICDP.

### Trial design {8}

The study has a two-by-two factorial, mixed methods design (see Table 1). The sample will consist of families with refugee background who have settled in Norway within the previous 9 years. Survey data will be collected pre-intervention (T1), mid-way through the intervention (T2), after the intervention (T3), and 1 year following intervention completion (T4). Qualitative data collection will begin after the families have completed the intervention.

**Table 1:** Study conditions

		A. Parenting program	B. Measurement feedback
	Condition 1	IY	MittEcho
	Condition 2	IY	None
	Condition 3	ICDP	MittEcho
	Condition 4	ICDP	None

#### Factor A: parenting program

Families will be randomized into either IY or ICDP parenting program at the beginning of each semester. Each parenting program is described in the “Intervention description {11a}” section.

#### Factor B: MFS

Group leaders who are using the MFS system (condition 1 and condition 3) will get weekly feedback based on the answers from parents in the MittEcho app. The feedback will be displayed graphically in a web-based login portal. Further description of MittEcho is found in the “Intervention description {11a}”section.

## Methods: participants, interventions, and outcomes

### Study setting {9}

Data for the present study will be collected from municipal agencies (e.g., refugee services, social welfare services) who work directly with parents in the target population. Sites will be recruited to join the study from all regions in Norway: Eastern, Southern, Western, Central, and Northern. The current list of participating study sites can be accessed on the project website: https://uit.no/research/pirm.

### Eligibility criteria {10}

Study participants will be recruited from several populations. The primary participants in the study are parents with a refugee background who have settled in a Norwegian municipality within the previous 9 years. They must also have at least one child between 6 and 12 years old. In order to participate in the study, the parents must be able to understand one of the eight study languages, which was chosen based on input from integration services in Norway: Norwegian, English, Arabic, French, Swahili, Turkish, Tigrinya, or Somali. Interview subjects are chosen with the desire for balance when it comes to interventions, age, gender, and background.

### Who will take informed consent? {26a}

Participants will be invited to join the study by local child welfare, municipal health stations, and municipal services for refugees. These services will identify potential participants based on inclusion criteria. Information will be given directly to parents by the services on behalf of the project with an invitation to participate in the study. Persons who are interested in participating will then return the signed consent to the project via electronic registration or postal service. All written and oral information is given in the families’ preferred available language. If necessary, a certified interpreter is used. The services will not give personally identifying information about potential participants to the project; however, we will get some information regarding the numbers of families invited so that we can estimate our response and recruitment rates.

Parents/guardians will give consent on behalf of their children. The children themselves will be given understandable information about the intent of the project and their participation in the interview. At the beginning of the interview, verbal assent from the children will be obtained. It will be emphasized that their participation is voluntary and that they can withdraw from the project at any point, even though their parents have given consent.

Parents and children in refugee families are considered a vulnerable group and the project is aware that there is a heightened responsibility to consider the well-being of vulnerable individuals that take part in research. Extra care will be taken in order to limit any detrimental effects on these individuals. The project will communicate that participation is voluntary and that there will be no negative consequences for participants who wish to withdraw from the project (or parts of the project), including that it will not affect their relationship with any services they are in contact with, or help received from different agencies (e.g., the services for refugees, health stations etc.).

### Additional consent provisions for collection and use of participant data and biological specimens {26b}

Biological specimens are not collected in this project. As of this time, there are no additional studies planned using this data. If additional studies are to be conducted that are not covered here, we would need to get updated consent forms.

## Interventions

### Explanation for the choice of comparators {6b}

Parenting interventions have been widely adopted in Norway to prevent violence towards children, to reduce child problem behaviors and promote positive parenting. The use of these interventions for families with refugee backgrounds is seen as a way to improve the integration process according to the immigration, integration, and child protection authorities in Norway. Both IY and ICDP are widely adopted throughout the country, but neither intervention has been evaluated for effectiveness for this population within Norway. Measurement feedback (MFS) is seen as a new way to improve treatment throughout a variety of mental health fields, though this trial is the first to evaluate an MFS for this population. In 2018, the national strategy for parental support in Norway was established: “Safe parents - safe children” The Government’s strategy for parental support (2018–2021) [18]. Based on this strategy, it has been decided that parent training programs are considered a mandatory part of the welcome/introduction program for refugees in Norway. Several programs have been identified for this use; the minority version of ICDP and the IY Basic Parenting programs are among those named in the report [19].

### Intervention description {11a}

The parenting interventions (factor 1) are both implemented as manual-based programs by their official program providers. Both IY and ICDP have extensive documentation which describes the length of the interventions and the contents of intervention meetings. Furthermore, over the course of intervention, data are collected from interventionists that document the content covered for each meeting session with the parents. The IY intervention in the PIRM-study is The School Age Basic Parenting program, which is conducted over 15 weeks with 3-h weekly meetings involving either one or both parents in a group-based setting. This program aims to “strengthen the parent-child interactions and attachment, reduce harsh discipline and foster parents’ ability to promote children’s social, emotional and academic development” (http://www.incredibleyears.com/programs/parent/school-age-basic-curriculum). This again aims to “prevent, reduce, and treat aggression and emotional problems in young children 0 to 12 years old” (http://www.incredibleyears.com/about/incredible-years-series/). The core components include content and process components. The core content components are as follows: positive parental attention, quality time with children, and sensitive responding to support the children’s development and a trusting relationship between parents and children. Development of predictable routines, positive limit setting, and handling of misbehavior are also important components. The content of the meeting is structured in different process core components with the collaborative process as the main element. Other process components are reflection and problem solving, short video vignettes of child-parent interactions, roleplays to practice skills learned, and home activities to enhance and generalize skills and knowledge developed in the group [20].

The minority version of the ICDP is conducted over 12 weeks with weekly meetings in 2 h sessions in a group-based setting. The program is intended to “support competence of care in parents and others who care for children in their profession” and “aims primarily to influence the quality of contact and the relationship between the child and the caregiver” (Children, Youth and Family Directorate, 2016, p. 15) [21]. The main goals of ICDP are as follows: (1) to promote the positive perception of the child, (2) to influence the caregiver's understanding of how important the interaction between the caregiver and the child is for the child's development, and (3) to promote the caregiver’s perception of herself/himself as a competent caregiver. ICDP consist of 5 core components: (1) caregivers perception of the child, (2) eight guidelines for good interaction with the child, (3) seven principles for sensitization used by a facilitator in relation to the caregiver, (4) six principles for implementation in meetings and in daily practice, and (5) adaptions to different groups and context. The content of the meetings is structured in different themes consisting of caregivers’ perception of the child and the eight guidelines for good interaction with the child. Home activities for the parents, short video vignettes, and roleplays to practice skills learned are used to stimulate reflection and discussion in the group.

The MittEcho feedback system (MFS; factor 2) was developed collaboratively for use in first-line, low threshold services. MittEcho feedback system consists of an app for entering data and a publication portal for viewing results. The MittEcho app is available in Google Play and Apple App Store. The parents in the MFS condition will answer questions based on their experience with parent training, the Measurement Feedback – Parent Scale questionnaire (see measures) and a self-evaluation of their progress on up to three goals that they choose themselves. Data based on parents’ replies to the app are uploaded and displayed in graphs accessible for group leaders in the MittEcho publication portal, which is stored on a secure data server (TSD). The results are updated weekly, and group leaders are expected to review and make evaluations of intervention progress for the group, as well as individual parents within the group.

### Criteria for discontinuing or modifying allocated interventions {11b}

There are no expected iatrogenic effects of participation. Parents are expected to complete the intervention unless they choose to withdraw.

### Strategies to improve adherence to interventions {11c}

All IY and ICDP group leaders undergo formal training for the interventions delivered by the respective program provider. They are given regular supervision by intervention experts. All group leaders are expected to fill out weekly checklists which ask them to evaluate their progress in the intervention. Group leaders in the MFS condition receive additional training by the members of the research team regarding how to access the MittEcho portal to view parents’ responses. They also receive general advice about how to interpret and act on the feedback for each of the participants and answer questions on the checklists regarding use of MFS. The families who complete the most measurement points for the MFS are entered in a drawing for movie tickets.

### Relevant concomitant care permitted or prohibited during the trial {11d}

Participants are not restricted from receiving other care related to parenting or mental health during the trial period.

### Provisions for post-trial care {30}

There is no expectation that the interventions are harmful to the parents. All of the parents in the study are covered under the Norwegian healthcare system, meaning that they have access to mental health resources should they be required.

### Outcomes {12}

The data will be obtained from parents (quantitative and qualitative), group leaders (quantitative and qualitative), teachers (quantitative), and children (qualitative) using web-based questionnaires at baseline, mid-intervention, post-treatment, and/or at 12-month follow-up. Interviews will be conducted in-person (where possible) to gather in-depth information about the parenting interventions. In addition, routine measurements are conducted weekly with help of the MittEcho app.

#### Quantitative surveys

The primary outcome measures note changes on: child problem behavior with the Eyberg Child Behavior Inventory (ECBI) [22], Parenting Practices Inventory (PPI) [23], and Parent Stress Index-Short Form (PSI/SF) [24] supported by qualitative interviews. The measures are available in multiple languages and evaluate parenting interventions and have been shown to be sensitive to change in families from a variety of contexts. Sum scores of the following scales from the quantitative surveys will be compared for the two factors (factor 1: parenting and factor 2: MFS) at T1 to assess possible baseline differences. Longitudinal analysis will follow and will compare changes in scores from, T1 to T2, T1 to T3, and T3 to T4 to assess differences in change for the two factors.

The ECBI is a well-validated, 36-item questionnaire that is scored by parents on frequency (1 yes or 0 no) and intensity (1 never to 7 always) of their child’s (2–16 years) problem behavior. High frequency scores (which range from 0 to 36) indicate more problem behaviors, while high intensity scores (which range from 36 to 252) indicates more intense problem. ECBI is widely used as a measure of problem behavior in studies of the IY program.

The Sutter-Eyberg Student Behavior Inventory-Revised (SESBI) is a teacher version of the ECBI scale. Like the ECBI, the SESBI is a report of the frequency and intensity of a child’s problem behavior.

The PPI is a survey of parenting behaviors and attitudes that are common for many parents [23]. The PPI consists of several sub-scales, of which we are using appropriate discipline, physical punishment, and monitoring.

The PSI/SF is a 36-item parent self-report of parent stress with 3 subscales (i.e., Parental Distress, Parent-Child Dysfunction, and Difficult Child). The items are rated from 0 strongly disagree to 4 strongly agree [24].

The resilience measure (READ) is a 39-item scale that was “…designed to assess the protective resources of personal competence, social competence, structured style, family cohesion and social resources to understand stress adaptation [25].”

#### Other quantitative measures

The Measurement Feedback - Parenting Scale (MF-PS) was developed to evaluate parents’ experiences in parenting interventions for families with refugee background. The survey is delivered via the MittEcho smartphone app.

The Intervention Goals (IG) are a list of 3 goals that parents choose to work on during the intervention. They then rate their satisfaction with progress on their goals over the course of the parenting sessions using the MittEcho smartphone app. In addition, user data for the use of the app and web-portal will be collected and analyzed.

#### Qualitative interviews

Children will be interviewed using techniques from The Dialogical Communication Method (DCM) [26] and the protocol for International Evidence-Based Investigative Interviewing of Children (NICHD) [27]. The interviews with parents and group leaders will be using semi-structured interview guides. The qualitative analyses are explorative, and in line with Denzin and Lincoln’s [28] description, different analytic strategies will be combined. All interviews will be analyzed using the interpretive research frame with a qualitative cross-sectional analytic strategy. A brief version of the interview guide for all participants is summarized in Table 2.

**Table 2:** Brief qualitative interview guide

	Background	Experiences with IY or ICDP	Refection concerning IY and ICDP	Effective components in IY and ICDP
**Parents**	Question about: country of origin, ethnicity, Refugee situation, parenting challenges, moving, resettle, acculturation, level of trust, and challenges as refugees.	Experiences regarding IY and ICDP	Reflection regarding IY and ICDP, support and helps when parenting in new context, and general impression of IY and ICDP	Questions about a group lesson, being parent and using IY or ICDP strategies.
**Children**	Question about a normal day, living in a new country, being a refugee, challenges, and strengths (important to balance the former question)	Question about the parents in Norway and country of origin: compare, identify the difference and giving examples of normal day with the parents	Reflection regarding: When are the parents at their best? Worse? “Wishful thinking”: What should change? What do they miss?	Question about if the parents use the IY and ICDP strategies with the children. “Happy thoughts”: What do the parents do as parents today, that you enjoy?
**IY group leaders**	Question about age, gender, education, years as IY or ICDP	Question about the IY or ICDP experiences.	Question regarding reflection on challenges, Comparison to other working methods, feedback form the families and if IY or ICDP are adequate	Question about what works or does not work in IY or ICDP, descend from the manual, ranking the parts of IY or ICDP from best to least favorite and argue for the ranking
**Video of IY groups**	Videos from IY: Looking for group dimension Individual behavioral Influence of the language interpreter	Looking for adherence to program implementation	Looking for feedback, disturbance, and extraordinary episodes	Consider the videos regarding different components of the manuals

Parent’s interviews will focus on investigating whether there is consistency between parent’s understanding of child rearing, care, and creating a safe environment for children. We also seek to identify children’s coping strategies and adaptation to a new context, as well as their experiences related to migration.

Group leaders’ experiences using MittEcho in this context and with this population will also be explored as part of the qualitative interviews and with particular interest in the usefulness of the system and process of using feedback.

Abductive reasoning will be used to analyze the qualitative interviews. Abductive reasoning is oriented to work from the data towards a theory [29]. As a qualitative research design, it works very well with evaluation of programs to “help increase the influence and impact of evidence-based prevention for population benefit” [30]. The chosen analytical strategies will contribute to the discussion of whether the program works, for whom, and under what conditions [31].

## Participant timeline {13}

### Enrollment

Rolling recruitment will be conducted throughout the course of the study period. Eligible families are identified by the municipalities and invited to attend the study. Families who sign and return the consent to the study office are considered enrolled in the study, where they are assigned a study ID and sent the pre-intervention questionnaire.

### Interventions

Interventions are delivered on a rolling basis, which corresponds to when the participants are recruited. The MFS intervention period overlaps directly with each of the parenting interventions. The IY parenting intervention takes place over 15 weeks, with additional weeks added for holidays as necessary. The ICDP parenting intervention takes place over 12 weeks, with additional weeks added for holidays.

### Assessments

#### Quantitative survey

The surveys are sent to the participants following enrollment in the study at pre-intervention (T1). Assessments are then sent (for parents only) mid-intervention (T2), which is 7 or 8 weeks after the IY groups start and 6 weeks after the ICDP groups start; at post intervention (T3) 15 weeks for IY, 12 weeks for ICCDP; and 1 year follow-up (T4). See Table 3 for a more detailed timeline of enrollment and data collection.

**Table 3:**
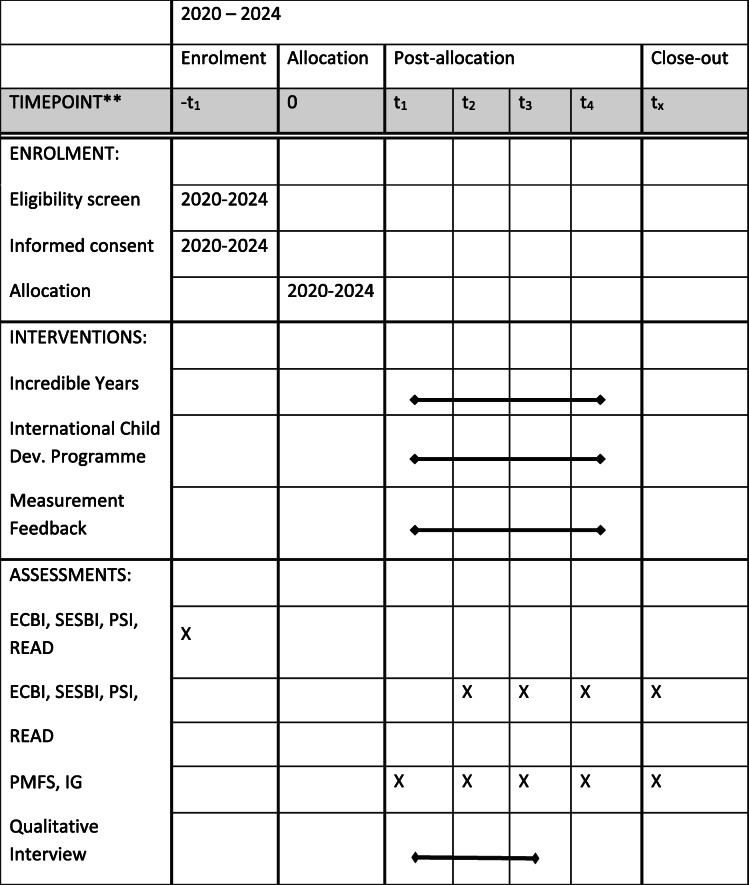
Study timeline

#### Qualitative interview

Interviews for the parents are conducted after the families are finished with the intervention (T3). Interviews for the group leaders are conducted after they have led at least one group to completion (T3). Interviews for the children are conducted after their parents have completed the group (T3 or later).

### Sample size {14}

#### Quantitative

The sample size for the PIRM study is based on a significance level of *p* = .05 and power *β* = 0.80. The expected effect size is Cohen’s *d* = .30 for the intervention groups based on differences between sum scores. In other words, we wish to detect a relatively small difference between enrollment in either of the parenting groups (factor 1) or in the addition of the MFS (factor 2). Based on this values, our aim is to recruit *N* = 360 families, *n* (IY, w/MFS) = 90, *n* (ICDP, w/MFS) = 90 , *n* (IY, no-MFS) = 90, n (ICDP, no-MFS) = 90. The study is powered to detect a relatively small effect size for main effects of either the parenting factor, IY_tot_ = 180 vs ICDP_tot_ = 180; or the MFS factor, MFS_tot_ = 180 vs no-MFS_tot_ = 180. Interaction effects between factors (e.g., IY w/MFS vs ICDP w/MFS) were not included in the power analysis due to the unknown nature of the main effects. With 6 families in each intervention group, the study would need roughly 5 municipalities to run two intervention groups, twice per year for 3 years.

#### Qualitative

The maximum target sample for the qualitative studies is as follows: *n* = 40 parents, *n* = 40 children, and *n* = 40 group leaders. The sample recruitment will cease when data saturation is reached, that is when additional interviews fail to provide novel information. The parents and children recruited for the qualitative study will be selected from a sub-set of the entire sample for the study (i.e., *N* = 360) based on their consent to be interviewed.

## Recruitment {15}

### Recruitment of municipalities

Recruitment of families will be conducted at the municipality level. Recruitment of municipalities began in 2019 with written invitations and information meetings which was held by the research staff in different sites all over Norway. An invitation letter was sent to the child and family services and immigrant services via the study coordinator, group leaders or Bufetat. The invitation letter described study aims, project participation, and both interventions. The recruitment process will be ongoing throughout the project period until the target of *N* = 360 families is reached

### Recruitment of families

Municipalities will identify and make initial contact with families who meet eligibility criteria. Each participating municipality, or site, is given some freedom for how they will make contact with eligible families. Some municipalities may opt to invite families to a meeting to learn about the study. Other families may be approached individually and recruited thusly.

#### Informed consent from participants

Families will be considered “recruited” when they have (A) returned a signed consent form electronically or via mail or (B) have submitted contact information electronically. In the latter case, these families’ data will be held only as long as it reasonably takes to receive a written consent. If no written consent is forthcoming, attempts will be made to contact these families to secure consent, but in the even that it is not possible, their data will be deleted, and a note will be made explaining the reason for our records. In the informed consent answered by parents, they will indicate whether their child can be contacted for an interview. In the event that the child may be contacted and agrees to the interview, recorded, verbal consent will be obtained by the researcher at the beginning of the interview. A copy of the information letter and consent form can be requested by email at: pirm@uit.no

#### Recruitment of group leaders

Group leaders are identified by municipalities and the implementation teams of IY and ICDP. If the group leaders require training, they will be trained by the implementation teams.

## Assignment of interventions: allocation

### Sequence generation {16a}

A project analyst at the primary site who does not have contact with sites or families will do the allocation of the factorial conditions using a random number sequence where groups at each site are assigned an ID and then each ID is assigned either to condition 1 (w/MFS) or 0 (no-MFS) (stage 1 randomization). A second list of IDs representing families is also randomized (stage 2) to condition 1 (IY) or 2 (ICDP). To understand the randomization, it is important to understand that each site will conduct both an IY and ICDP parenting group concurrently, so that families can be randomly assigned to either group. However, sites are not capable of running four unique groups concurrently (i.e., IY w/MFS, IY no-MFS, ICDP w/MFS, ICDP no-MFS). Therefore, randomization occurs in two stages. Stage 1 randomly assigns the two groups at each site to the MFS condition (factor 2). For example, at site 1, the IY group is randomly assigned to the MFS condition, and the ICDP group is assigned to the no-MFS condition. The decision to randomize the MFS condition at the group level is practical: in order to use the MFS properly, the group leaders need to respond to the feedback. Therefore, any actions taken by the group leader will affect the entire group. In order to minimize systematic differences that may be introduced by group leaders, the groups will be re-randomized to the MFS condition when a new group begins, or roughly once per semester.

Stage 2 randomization is responsible for assigning families at each site to one of the two parenting conditions (i.e., IY or ICDP; factor 1). Stage 2 randomization by the primary site will occur after the T1 survey has been completed by the participating families.

### Concealment mechanism {16b}

Families, group leaders, and implementation teams will be unaware of their treatment condition until after they have completed the T1 survey.

### Implementation {16c}

To preserve the factorial design, we need to randomize to four conditions (see table in the “Trial Design” section of this document). We cannot randomize families to four separate conditions at each site, because this would require running four groups at each site every semester (see also the “Sequence generation {16a}” section). It would not be feasible for the municipalities to conduct four groups in parallel due to the staffing requirements. Therefore, randomization in PIRM has two stages: stage 1, randomization to MFS condition at the group level, and stage 2, randomization of families to IY or ICDP parenting groups.

Recruitment of families at each site will be ongoing. Families will be randomized when (a) a maximum of 12 families are recruited or (b) a cutoff date is reached each semester. The cutoff date will be established by the amount of time it takes to complete a group, (i.e., 15 weeks for IY, 12 weeks for ICDP). In Norway, summer holidays usually occur between June and August; therefore, groups must have adequate time to finish within the time period from January to June or late August to December.

The parenting groups (2 per site) are randomly assigned to one of the MFS (Y/N) conditions so that one group at each site will be in the MFS conditions. For example, at site 1, the ICDP group is randomly assigned to the MFS condition during the first semester. The following semester the groups will be reassigned and the IY group at site 1 may end up in the MFS condition.

## Assignment of interventions: blinding

### Who will be blinded {17a}

Because of the nature of behavioral interventions, blinding the participants to condition is impossible once the groups start, i.e., they will know if they are in IY or ICDP. Researchers and analysts will not have contact with the families and will not have access to the key file with names and contact information of individual families. All analyses of quantitative data will be conducted using files with participant IDs and intervention condition dummy codes.

### Procedure for unblinding if needed {17b}

Families will know their own condition once the interventions begin; therefore, unblinding is irrelevant.

## Data collection and management

### Plans for assessment and collection of outcomes {18a}

All project data will be stored and analyzed on a platform developed at the University of Oslo (UiT), the Services for Sensitive Data (TSD). TSD is designed to be a secure platform for storing sensitive data and also to process/analyze data using a remote desktop connection.

### Quantitative surveys

#### Families/parents

After families have been recruited, they will receive links to the online surveys. The surveys can be filled out electronically on the participant’s smartphone, desktop/laptop, or tablet. The survey data are delivered via Nettskjema and data are stored in TSD. Data are collected at four measurement points (pre/T1, mid/T2, post/T3, and follow-up/T4). Data from children or parents who withdraws consent will be deleted.

#### Group leaders

Group leaders will be asked to fill out an online survey prior to group start. The group leaders are also requested to fill out weekly checklists regarding their experiences using the intervention which details the content that they cover and use of the MittEcho feedback system. Parent’s attendance is also registered in the checklists. After the group leaders have had experience using the intervention, they will be asked to fill out the group leader questionnaire (GLQ).

#### Teachers

Teachers will receive an invitation to fill out a survey about the child from families participating in the study if the parents have consented. If more than one child in the specific class is participating, the teachers complete one survey for each child. Data is collected at the same measurement points as parent reports (pre/T1, post/T3, and follow-up/T4).

### MFS/MittEcho

MFS data are collected weekly for families in the MFS conditions. The MittEcho app is used to enter the MFS data directly via smartphone or tablet. Data are automatically uploaded to TSD.

### Qualitative interviews

All qualitative interviews will be conducted in-person with parents and children and in-person or over video conference with the group leaders. Data will be collected using smartphones as recording devices that upload the encrypted audio files directly to TSD. The audio recordings will be transcribed in situ on TSD by professional transcribers for analysis.

### Plans to promote participant retention and complete follow-up {18b}

We intend to maintain contact with participating families using the local services. Participants will be compensated for their participation at post- (T3) and follow-up (T4) with gift cards.

## Data management {19}

Data in the PIRM study are considered sensitive and will be gathered and handled following strict guidelines. All data will be stored in a centralized, encrypted database at the University of Oslo which has been developed for the purpose of secure data storage. The database which stores the data for the project is called TSD. It is accessible only to a few personnel who can be given granular access by the project leader to areas that are relevant only to them.

### Quantitative surveys

The quantitative data will be gathered using an online survey developed in Nettskjema. All data are stored on an encrypted, secure database at the University of Oslo. Access to the data files are restricted to project staff and must be approved by the project leaders.

### MFS/MittEcho

MittEcho app data are uploaded directly in encrypted form to TSD on a weekly basis. App data are then directed to a web based MittEcho portal for only approved members of the project. Interventionists in the study apply for membership and gain access to the MittEcho results by secure, two-factor log in. Raw data are stored in TSD until used for analysis.

### Qualitative interviews

The qualitative data will be gathered using an app (Nettskjema Dictaphone App) that automatically uploads the audio files to TSD. The audio files will then be accessible to researchers and transcribers using a remote desktop to access files on TSD.

### Monitoring consort statement

The consort statement is updated throughout the study to track inclusion into, exclusion from, and dropouts from the study. The consort is stored and accessible on TSD. All cases screened are (1) reported to the local coordinator and (2) entered into the “Consort statement” registration by project staff.

#### Drop out during or following randomization

Participants who drop out during or following randomization, i.e., do not wish to continue in the intervention, may be asked why, though they will be reminded that no reason is necessary to drop out. If the participant does not want to participate in the PIRM follow-up, this is registered in the study database.

## Confidentiality {27}

Collected data will be stored on TSD secured storage in files using unique IDs that are linked to the respondents using a matching key that will be stored separately. The matching key will only be accessible to the project coordinators. Project scientists and analysts will only have access to files that use the unique ID. In addition, results of the study will primarily be reported in aggregate. If an individual case or cases are reported (e.g., quotes from qualitative interviews), directly and indirectly identifying information will be omitted. Data will be stored long term in deidentified form.

## Plans for collection, laboratory evaluation, and storage of biological specimens for genetic or molecular analysis in this trial/future use {33}

Biological specimens are not being collected in this study.

## Statistical methods

### Statistical methods for primary and secondary outcomes {20a}

We will conduct analyses in a longitudinal framework, controlling for scores at baseline. For example, to test the primary outcomes of the effectiveness study, we will use linear mixed modeling to assess changes in parenting or child behavior in the different intervention groups while controlling for scores pre-intervention. The analyses will account for independence within the respondents (time as a random effect) as well as group membership, because the randomization to the MFS condition is at the group level. We will also look at interactions between main effects given enough power, for example, between the MFS conditions (factor 2) and the parenting conditions (factor 1).

See the “Outcomes {12}” section for description of qualitative analysis.

### Interim analyses {21b}

No plans for interim analyses exist at this time.

### Methods for additional analyses (e.g., subgroup analyses) {20b}

Sub-groups analyses will be performed in a mixed methods modeling framework. Some of the sub-groups analyses will be determined based on factors at baseline, such as language or country of origin. Actual sub-groups will be dependent on the characteristics of the recruited sample.

### Methods in analysis to handle protocol non-adherence and any statistical methods to handle missing data {20c}

We will analyze the quantitative data in an ITT approach. We intend to use full information estimates (e.g., maximum likelihood) or multiple imputation to include cases that may have missing data [32, 33]. This approach introduces less bias into analyses than other approaches (e.g., listwise deletion, regression imputation, etc.).

### Plans to give access to the full protocol, participant level-data, and statistical code {31c}

Project coordinators and scientists will have access to the data via remote desktop environment in TSD. The data will be anonymized by removing secondarily-identifying information after the study and research period is ended. This dataset will be made public in accordance with research practices at UiT The Arctic University of Norway.

## Oversight and monitoring

### Composition of the coordinating center and trial steering committee {5d}

Data will be monitored by staff at the host organization and the researchers on the study. Regular examination of the data will be conducted to assess integrity and address possible data collection issues.

### Composition of the data monitoring committee, its role and reporting structure {21a}

The DMC will be comprised of people who are internal and external to the project; however, all will be employed with the host organization or its project collaborators. This team will be responsible for maintaining data integrity and reporting anomalies to the project group.

### Adverse event reporting and harms {22}

The interventions in this study are not expected to do harm; however, all interventionists are part of the mental healthcare system and have local protocols to which they must adhere in the course of their work.

### Frequency and plans for auditing trial conduct {23}

There is no plan for auditing trial conduct outside of the existing systems that are responsible for audits, such as funders or ethics committees.

### Plans for communicating important protocol amendments to relevant parties (e.g., trial participants, ethical committees) {25}

Changes to the protocol are communicated to the funding agency as they occur and during our annual reports. Protocol changes that are directly relevant to participants are communicated through the contact personnel at the participating research sites, as they have direct contact with the participants.

### Dissemination plans {31a}

Project results will be communicated through scientific conferences; peer reviewed, scientific journal articles; popular publications; project and affiliated webpages; and Ph.D. dissertations. As part of the agreement for grant funding, all scientific journal publications will be open access.

## Discussion

The PIRM study takes place within the context of recent requirements for refugee families to participate in parenting programs. The knowledge gained regarding effects of the parenting interventions will further inform policymakers regarding the use of these programs for families with refugee background. Our results will further provide guidance about the use of a weekly measurement and feedback within the context of these interventions. This guidance will evaluate the effectiveness of MFS with parenting interventions, as well as its feasibility of implementation in first-line preventive services.

## Trial status

This is the 2nd version of the study protocol, completed in February 2020. The first participants were recruited in September 2020. Recruitment will continue until the second half of 2023.

Contact for the public and scientists regarding the status of the study can be addressed to Associate Professor Lene-Mari P. Rasmussen at telephone number +47 776 44 000 or via email at PIRM@uit.no. The mailing address for the project is as follows: UiT Norges arktiske universitet, Det helsevitenskapelige fakultet, RKBU Nord, Varemottak-MH, plan 6, Sykehusv. 44, 9019 TROMSØ.

## Abbreviations

ECBI: Eyberg Child Behavior Inventory
ICDP: The International Child Development Program
IG: Intervention Goals
IY: The Incredible Years Basic Parenting Program
MFS: Measurement and Feedback System
P-MFS: Parenting Measurement and Feedback Scale
PPI: Parenting Practices Inventory
PSI: Parent Stress Index-Short Form
READ: Resilience measure
RKBU: The Regional Center for Child and Adolescent Mental Health and Child Welfare
SESBI: Sutter-Eyberg Student Behavior Inventory-Revised
TSD: Services for Sensitive Data, University of Oslo
